# Supplementary material for: Availability of Ferritin-Bound Iron to Enterobacteriaceae
Source: Int J Mol Sci. 2022 Oct 28;23(21):13087. doi: 10.3390/ijms232113087 (PMC9657528; doi:10.3390/ijms232113087)
Supplement: Supplementary file 1 [file ijms-23-13087-s001.zip › Figure S1.pdf]

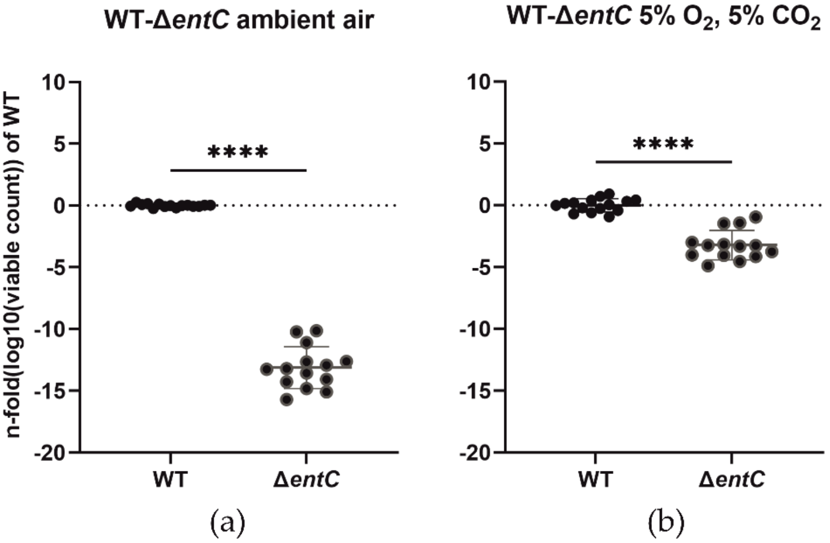

Figure S1. *S.*Tm ATCC 14028 and a derivative Δ*entC* mutant were grown in IMDM (a) in ambient air (N=15, n=5) or (b) in 5 % O_2, 5 % CO_2 atmosphere (N=15, n=5). Growth data were logarithmized and then normalized to the WT. Graphs are depicted as mean ± SD. For statistical analysis Student's t-test was used (****: $p<0.0001$). N: number of biological replicates, n: number of experiments conducted, WT: wildtype, Δ*entC*: *S.*Tm enterobactin/salmochelin knockout mutant strain
