# Supplementary material for: Availability of Ferritin-Bound Iron to Enterobacteriaceae
Source: Int J Mol Sci. 2022 Oct 28;23(21):13087. doi: 10.3390/ijms232113087 (PMC9657528; doi:10.3390/ijms232113087)
Supplement: Supplementary file 1 [file ijms-23-13087-s001.zip › Figure S2.pdf]

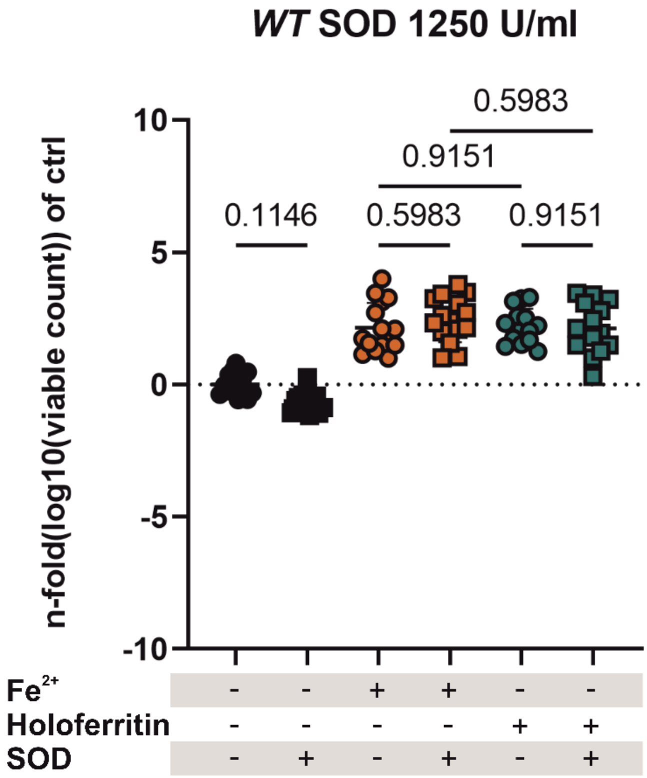

Figure S2. Iron-dependent growth of WT *S.*Tm (N=15, n=5) with or without SOD, depicted as squares and circles, respectively (N=15, n=5). Cultures were incubated at 5% O_2 5% CO_2, 37°C and 200 RPM. Growth data were logarithmized and then normalized to the corresponding control without any supplement. Expression data were normalized to their corresponding controls: no supplement (circles) or SOD (squares). Graphs are depicted as mean ± SD. For statistical analysis one-way ANOVA corrected for multiple comparisons with the Holm-Sidak post hoc test was used (numbers indicate a non-significant p-value > 0.05). N: number of biological replicates, n: number of experiments conducted. SOD: superoxide dismutase, WT: wildtype *S.*Tm.
