# Supplementary material for: Availability of Ferritin-Bound Iron to Enterobacteriaceae
Source: Int J Mol Sci. 2022 Oct 28;23(21):13087. doi: 10.3390/ijms232113087 (PMC9657528; doi:10.3390/ijms232113087)
Supplement: Supplementary file 1 [file ijms-23-13087-s001.zip › Figure S3.pdf]

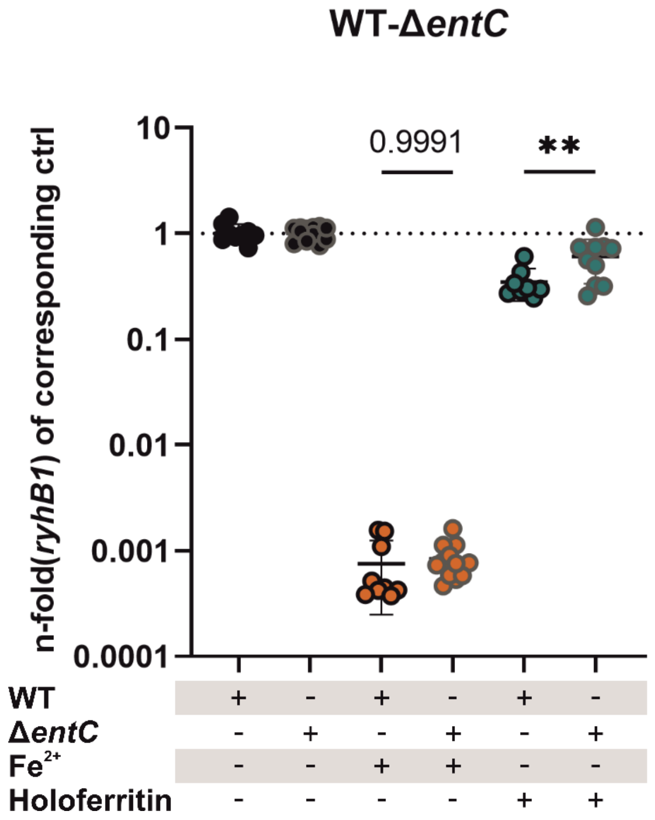

Figure S3. Fold change in *RyhB1* sncRNA expression normalized to the mean of the corresponding iron free control of WT *S.*Tm (N=9, n=3) and the Δ*entC* strain (N=12, n=4) upon different iron sources. Expression data were normalized to their corresponding controls: WT (black circles) or Δ*entC* strain (grey bordered black circles). Gene expression was normalized using the ΔΔct method using the *16S* ribosomal RNA (*16S rRNA*) and DNA-dependent RNA polymerase (*rpoB*) mRNA as reference transcripts. Graphs are depicted as mean ± SD. For statistical analysis one-way ANOVA (a-d) corrected for multiple comparisons with the Holm-Sidak post hoc test was used (**: $p<0.01$, numbers indicate a non-significant p-value > 0.05). N: number of biological replicates, n: number of experiments conducted. WT: wildtype *S.*Tm, Δ*entC*: *S.*Tm enterobactin/salmochelin knockout mutant strain.
